# FRET microscopy autologous tumor lysate processing in mature dendritic cell vaccine therapy

**DOI:** 10.1186/1479-5876-8-52

**Published:** 2010-06-03

**Authors:** Laura Fiammenghi, Valentina Ancarani, Tilman Rosales, Jay R Knutson, Massimiliano Petrini, Anna Maria Granato, Elena Pancisi, Laura Ridolfi, Ruggero Ridolfi, Angela Riccobon, Paolo Neyroz

**Affiliations:** 1 Immunotherapy and Somatic Cell Therapy Laboratory, Istituto Scientifico Romagnolo per lo Studio e la Cura dei Tumori (I.R.S.T.) Meldola, Italy; 2 Laboratory of Molecular Biophysics, National Heart, Lung and Blood Institute, National Institutes of Health, Bethesda, USA; 3 Department of Biochemistry "G. Moruzzi", University of Bologna in Rimini, Italy

## Abstract

**Background:**

Antigen processing by dendritic cells (DC) exposed to specific stimuli has been well characterized in biological studies. Nonetheless, the question of whether autologous whole tumor lysates (as used in clinical trials) are similarly processed by these cells has not yet been resolved.

**Methods:**

In this study, we examined the transfer of peptides from whole tumor lysates to major histocompatibility complex class II molecules (MHC II) in mature dendritic cells (mDC) from a patient with advanced melanoma. Tumor antigenic peptides-MHC II proximity was revealed by Förster Resonance Energy Transfer (FRET) measurements, which effectively extends the application of fluorescence microscopy to the molecular level (<100Å). Tumor lysates were labelled with Alexa-488, as the donor, and mDC MHC II HLA-DR molecules were labelled with Alexa-546-conjugated IgG, as the acceptor.

**Results:**

We detected significant energy transfer between donor and acceptor-labelled antibodies against HLA-DR at the membrane surface of mDC. FRET data indicated that fluorescent peptide-loaded MHC II molecules start to accumulate on mDC membranes at 16 hr from the maturation stimulus, steeply increasing at 22 hr with sustained higher FRET detected up to 46 hr.

**Conclusions:**

The results obtained imply that the patient mDC correctly processed the tumor specific antigens and their display on the mDC surface may be effective for several days. These observations support the rationale for immunogenic efficacy of autologous tumor lysates.

## Background

Dendritic cells (DC) are the most potent leukocyte populations which control the primary immune response [1]. As antigen-presenting competent cells, they recognize and process antigens in the peripheral blood and tissues, migrate to draining lymph nodes, and finally present antigens to the target resting lymphocytes. Antigens are very efficiently internalized and processed by immature DC (iDC), but to achieve a productive T-cell response iDC must differentiate to mature DC (mDC), which express high levels of the cell-surface antigen-bearing major histocompatibility complex, class II (MHC II). In the multifaceted set of relationships that exist between the immune system and cancer, therapeutic vaccination has been accepted as a valid approach to overcoming the established state of immunotolerance between the two systems [2,3]. The use of DC, derived from peripheral blood precursors and pulsed with tumor antigens, forms the basis of experimental and clinical trials on anti-tumor vaccinations [4,5]. Although overall response rates for vaccination are still somewhat limited, results obtained with DC vaccinations can be considered a very promising therapeutic strategy [6]. To refine the implementation of this approach, evaluation of both the DC migration activity to lymphatic tissues, and the correct presentation of tumor antigens in MHC II complexes at the DC membrane surface, is of critical importance. From this perspective, translational work to link the results from studies at the cellular and molecular level with those from clinical investigations is of great interest.

In a previous report, *in vivo *DC migration was investigated within the context of a clinical trial of anti-tumor vaccination [7]. In particular, it was shown that mDC exhibit a six- to eightfold higher migration rate than iDC. Following that study, here we have investigated the molecular traits of the MHC II complexes of DC from a melanoma patient pulsed with autologous whole tumor lysate (ATL).

Our aim was to show that the autologous lysate (diverse, in character or content of tumor specific antigens, from isolated peptides, but representing the real "drug") could be processed by the patient's DC and loaded on the membrane surface as MHCII complexes, a crucial information for the clinical evaluation of patients involved in vaccination therapy trials.

## Methods

### Dendritic cells

Dendritic cells (DC) were prepared as described previously [6]. Peripheral blood monocytes obtained by leukapheresis without previous mobilization were purified on Ficoll-Paque gradients (Ge Healthcare Milan, Italy), incubated in tissue culture flasks with CellGro DC medium (Cell Genix, Freiburg, Germany) at a density of 10^7 ^cells/ml for 2 hr, and the adherent cells were incubated in CellGro DC medium containing 1000 IU/ml rhIL-4 and 1000 IU/ml rhGM-CSF (Cell Genix, Freiburg, Germany) for 7 days. On day 6, the DC culture was pulsed with autologous tumor lysate (ATL) (100 μg/ml). On day 7, the cells were defined as immature DC (iDC). After eliminating the previous culture medium, pulsed iDC were cultured for a further 2 days with a cocktail of cytokines (TNFα, IL-1β, IL-6, Cell Genix, Freiburg, Germany; Prostin E_2_, Pfizer, Puurs, Belgium). On day 9, the cells were defined as mature DC (mDC). iDC and mDC phenotypes were determined by single or two-color fluorescence analysis.

### ATL preparation and labeling

Surgically removed tumor samples were mechanically and enzymatically dispersed to create a single-cell suspension in RPMI 1640 (PAA Laboratories GmbH, Pasching, Austria) and the tumor lysate was prepared as described previously [6]. Protein concentrations were determined and aliquots were stored at -80°C until use. For fluorescence labeling, ATL was reacted with Alexa Fluor^®^-488 succinimidyl ester (Molecular Probes-Invitrogen, USA) according to the supplier's instructions. Size-exclusion chromatography on Sephadex G-25 *superfine *(Ge Healthcare Milan, Italy) was used to separate the bound from the free dye. Analytical sodium dodecyl sulphate polyacrylamide gel electrophoresis (SDS-PAGE) on gradient (4%-20%) was performed to evaluate the protein content of ATL and the goodness of the fluorescence labeling.

### Immunofluorescence

Cells (3 × 10^5^), pulsed with ATL-Alexa488 (80 μg/10^6 ^DC), were plated on coverslips pretreated with poly-D-lysine (Sigma Milan, Italy) and fixed with several drops of cold methanol at -20°C for 2 min. Cells were stained with mouse monoclonal HLA-DR (HL12) primary antibody (Santa Cruz, CA, USA) (1:100), followed by Alexa Fluor^®^-546 goat anti-mouse IgG (Molecular probes, Invitrogen) (1:2000).

### Confocal FRET measurements

Image acquisition and FRET efficiency by acceptor photobleaching measurements [8] were performed using a Leica TCS SP5 equipped with an argon ion and a DPSS laser with output lines at 488 nm and 561 nm, respectively. All samples were imaged with a Leica Plan Apo 63 × 1.4 oil immersion lens. FRET was resolved from the increase of donor fluorescence in the bleached region of interest (ROI). Data analysis was accomplished by the Leica software application for acceptor photobleaching, and the energy transfer efficiency was calculated according to equation 1 as:

where D_*postbleach *_is the fluorescence intensity of the donor after photobleaching and D_*prebleach *_is the fluorescence intensity of the donor before photobleaching. As a control, non-bleached areas were also analyzed for FRET. Before- and after-photobleaching, images were acquired by simultaneous excitation with 488 nm and 561 nm laser lines at 5% and 9% of the total power intensity, respectively. Photobleaching was obtained by scanning in a zoomed region, over six vertical *Z *sections, with the 561 nm excitation laser line at 100% of its power intensity.

## Results

### Immunofluorescence

**Figure 1:**
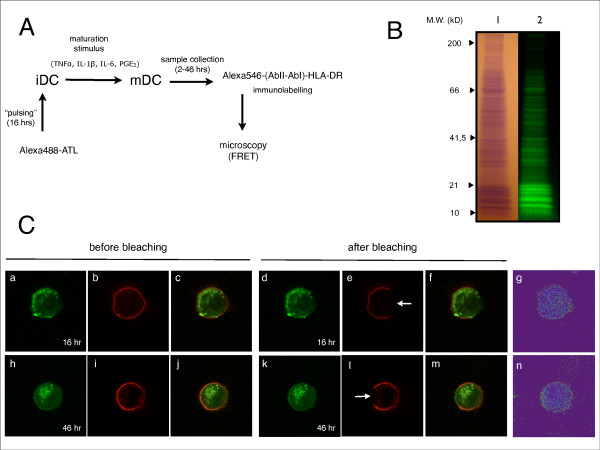
**Experimental plan and fluorescence images**. (A) Scheme of the experimental plan. (B) SDS-PAGE analysis of ATL. Proteins gel electrophoresis separation was run on acrylamide gradient (4%-20%). The ATL sample is shown after Coomassie brilliant Blue staining of the proteins bands (lane 1) and by UV transillumination, before staining of the proteins bands, to reveal the extent of the fluorescence labelling (lane 2). (C) FRET analysis of mDC loaded with Alexa488-labeled ATL and immunolabelled with HLA-DR(HL12) mAb and Alexa546-conjugated IgG. The upper panels refer to the sample analyzed at 16 hours after the maturation stimulus and the lower panels refer to the sample analyzed at 46 hours after the maturation stimulus. Panels are divided in sets of images acquired before and after acceptor photobleaching (see Materials and Methods). Donor images were acquired in the green channel (a, h, d and k) and acceptor images were acquired in the red channel (b, i, e and l). White arrows (e and l) indicate the bleached regions. The relative merged images are also shown (c, j, f and m). FRET efficiency was calculated using eq. 1 and the results are presented as pseudo-color images (g and n).

The tight regulatory control of peptide-MHC II complex formation in DC have been dissected and clearly described in prior fundamental biological studies [9-11]. In particular, it has been shown that effective presentation of peptide-MHC II complexes requires DC maturation and that this final differentiation is a major control in priming T cells *in vivo*. Due to the impact of this finding on the optimal use of DC in cancer immunotherapy, as an adjunct to a phase I/II clinical trial on advanced melanoma patients we explored the potential transfer of ATL peptides to MHC II complexes at the DC plasma membrane as a function of time after maturation. In Figure 1A a summary scheme of the experimental plan is presented. iDC were pulsed with Alexa488-labeled ATL for 16 hr and, after the wash out of lysate, matured with a standard cytokine cocktail (see Methods). At increasing times (2-46 hr) from the maturation stimulus, mDC HLA-DR molecules were immunolabelled with Alexa546-bioconjugated IgG, and double fluorescence stained cells were analyzed by confocal microscopy to reveal FRET. In Figure 1B the gel electrophoresis analysis of a typical ATL - Alexa488 labeling procedure is shown, while in Figure 1C the characteristic mDC images obtained at 16 hr and 46 hr after the maturation stimulus are presented.

### FRET measurements

In FRET experiments a *donor *and an *acceptor *are defined by the overlap between the emission spectrum of the first and the excitation spectrum of the second. An excited donor will return to the ground state through an acceptor *via *FRET provided that the acceptor molecule is in close vicinity (<~80Å). In our experimental design, Alexa488-ATL molecules represent the *donor *and Alexa546-(AbII-AbI)-HLA-DR represent the *acceptor*. Under these conditions, detection of FRET is an accurate signature of proximity (requiring physical interaction) between ATL peptides and HLA-DR molecules. The efficiency of FRET is strongly distance dependent, so overall FRET efficiencies will have an upper limit set by the distance of closest approach between the ATL dye and the dye on the secondary antibody.

**Figure 2:**
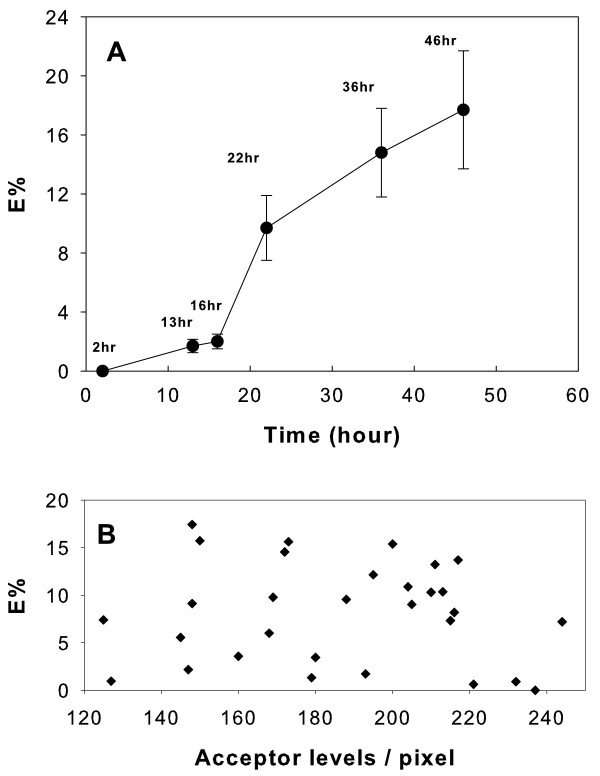
**FRET measurements**. (A) Averaged FRET efficiency of mDC as a function of time from the maturation stimulus. The data and the Standard Errors (±SE) refer to FRET measurements performed over at least three fields for each sample (n = 3-5) and different ROIs (n = 30-55) inside the bleached regions. The *x *axis displays the time in culture after maturation stimulus. (B) Plot of the independence of E% from acceptor levels. The data shown were generated from image measurements 22 hr after maturation. The acceptor levels refer to the intensity of the image acquired before acceptor photobleaching and analyzed *versus *the recovered E%, on a pixel by pixel basis.

FRET efficiency measurements obtained for mDCs at 16 hr and 46 hr after the maturation stimulus are presented in Table 1. The overall FRET efficiency of mDC examined 46 hr after maturation was significantly higher than that measured after 16 hr. In the table this evidence is clearly indicated by the changes in intensity levels of the Donor Pre and the Donor Post columns, respectively. We confirmed this observation by studying the FRET efficiency of mDC for 2 hr to 46 hr after the maturation stimulus (Figure 2A). Although HLA-DR molecules were found concentrated at the dendritic cell plasma membrane even shortly after maturation (≤4 hours, data not shown), no significant proximity with the ATL antigens was detected up to 16 hr. However, from 16 to 22 hr, a steep increase of FRET was revealed, and the trend continued upwards up to 46 hr. These results suggest that specific tumor antigen peptides are transferred to MHC II complexes, and that this process is significant 22 hr after maturation. Moreover, the data also confirms that antigen presentation is still fully effective after 46 hr.

**Table 1:** mDC FRET efficiency measured 16 hr and 46 hr after maturation stimulus

mDC 16 hr	ROI 1	ROI 2	ROI 3	ROI 4	ROI 5	ROI 6
**Donor Pre**	33	23	26	25	29	46
**Donor Post**	34	23	25	26	28	45
**Acceptor Pre**	46	59	60	47	35	74
**Acceptor Post**	8	8	7	6	33	72
**E (%)**	2.94	0	0	3.85	0	0

**mDC 46 hr**	**ROI 1**	**ROI 2**	**ROI 3**	**ROI 4**	**ROI 5**	**ROI 6**

**Donor Pre**	31	45	43	41	37	44
**Donor Post**	36	62	52	49	37	42
**Acceptor Pre**	56	142	132	142	139	133
**Acceptor Post**	7	9	8	8	141	139
**E (%)**	13.9	27.4	17.3	16.3	0	0

ROI, Region of interest; Pre, before photobleaching; Post, after photobleaching; mDC 16 or 46 hr, Dendritic Cells matured for 16 or 46 hr; E (%), Energy Transfer Efficiency. The data shown refer to six significant ROIs of the experiments reported in Figure 1A. ROI1 to ROI4 represent regions selected within the bleached area, whereas ROI5 and ROI6 represent regions selected outside the bleached area, which were used as controls. The numbers in the "Donor" and the "Acceptor" rows indicate the fluorescence intensity levels detected in each ROI.

### FRET efficiency analysis

It should be noted that in extremely (donor or acceptor) overloaded cells, FRET might sometimes be detected as a result of accidental proximity due to surface density-dependent interactions [12-14]. To be certain our FRET results heralded true proximity, we tested FRET levels *versus *loading on a pixel by pixel basis. Any "artifactual" FRET from overloading should be strongly correlated with levels per pixel. Under our experimental conditions, the total tumor lysate fluorescence (donor), complexed at the mDC membrane surface, should be intrinsically depleted due to the intracellular antigen degradation and processing events. For this reason, we assumed that only the MHC II molecules (acceptor) could represent a source of artifactual FRET.

Figure 2B shows a plot of the acceptor levels *versus *FRET efficiency. This analysis strongly indicates that E% is independent of acceptor levels, and thus negates interpretations that sever the link between FRET efficiency and tumor lysate peptide-MHC II proximity.

## Discussion

Proof of the specific activation of immune responses is crucial in the overall rationale of cancer immunotherapy, and, more specifically, it is needed to convincingly address any analysis of immunogenic efficacy. In the present work we evaluated the presentation of ATL peptides onto MHC II of mDC from a patient with advanced melanoma.

The pattern of the protein content of ATL has been presented in Figure 1B together with the result of its homogeneous fluorescence labeling. These products were used previously to monitor the uptake and the processing of ATL in DC by fluorescence microscopy imaging [15]. Here, the antigens (ATL) and antigen-capturing molecules (MHC II) were tagged to act as donor-acceptor pairs, and FRET measurements were performed to resolve the physical interactions between ATL and MHC II. Under these conditions, our results indicate a significant correlation between FRET efficiency and the time after maturation stimulus (Figure 2A). This observation is consistent with an increasing transfer of ATL peptide-loaded MHC II molecules on the mDC membrane. This process is significant 22 hr after maturation, and antigen presentation remains fully effective after 46 hr. The kinetic response observed is in excellent agreement with those reported on the transport of specific HEL-peptide-MHC II complexes at the DC surface [9], and the accumulation of MHC II complexes on mDC induced by inflammatory stimuli [10]. Yet, in accord with these reports, the apparent discrepancy between the high levels of acceptor fluorescence and the absence of FRET detection shortly after maturation (≤4 hours), could possibly be related to the rapid turnover of unloaded MHC II molecules observed in developing DCs.

In Figure 2B we addressed the potential effects of MHC II density over FRET by plotting acceptor levels *versus *the efficiency, E%. This test was developed to study the distribution of proteins at the apical surface of MDCK cells [14]. In particular, in the appendix of that survey, the theoretical dependence of FRET was separated into random or clustered distribution of donor- and acceptor-labeled molecules. It was clearly shown that the clustered model predicts that the efficiency will be independent of the surface densities of the labeled molecules. As mentioned above, given that newly synthesized class II molecules are produced in increased amounts in the first 24 hours after maturation [10], in our study we were particularly cautious about the FRET detection bias due to acceptor *overcrowding *[12]. In this respect, a more distinctive feature of MHC II organization on the plasma membrane of DC was elucidated recently by Unternaehrer and coworkers [16] in which MHC II molecules were found to cluster by a lateral association mediated mechanism.

In our study, the independence of E% from acceptor levels (random distribution of E%) clearly indicates the absence of a nonspecific density contribution to FRET and fits the clustered model. Thus, we assign the significant increase of FRET efficiency, observed at the membrane surface as a function of time from maturation, to the actual transfer of specific tumor antigen peptides into MHC II clustered complexes.

This single-case survey on an advanced melanoma vaccination trial shows that autologous tumor lysates are correctly processed and presented at the mDC membrane surface in melanoma patients. In addition, this time-dependent profile is consistent with a delayed mDC antigen display, a property that is crucial for their role in vaccination-triggered immune surveillance [17]. Yet, the methodology described and the parameters obtained (i.e. FRET signals) can be applied to follow-up studies to analyze and evaluate their prognosis value in addressing the efficacy of immunotherapy protocols.

Finally, it is worth commenting on the potential wealth of information that could be gleaned from FRET measurements when maximal FRET efficiency is known. In favorable circumstances, a quantitative data analysis approach is possible (i.e. a measure of the absolute changes in the amounts of antigen-loaded MHC II molecules at the DC membrane surface). Unfortunately, this information can only be obtained from extensive studies where appropriate standards are available (i.e. oligonucleic acid hybrids, streptavidin-biotin coupled donor-acceptor pairs) [18], or when specific tagged molecules can be engineered [12]. Under our particular experimental conditions, we could not define the maximal FRET efficiency of the investigated donor-acceptor system (Alexa488-ATL - Alexa546-(AbII-AbI)-HLA-DR). Additional "semi-quantitative" data interpretation would be affected by large approximations, and would also rely on uncertain assumptions. Nonetheless, the measured relative changes of FRET efficiency with time from maturation are intrinsically significant and relevant for the clinical evaluation of immunotherapy vaccination trials.

It has to be pointed out that we chose the acceptor photobleaching FRET method for its complete insensitivity to certain artifacts, including the direct excitation of acceptor. According to this FRET measurement method, both of the images (i.e. the green and in the red channels) were acquired before and after photobleaching through the appropriate emission barrier filters. Moreover, for each sample, three different staining preparations were carried out: +Alexa488 -Alexa546; +Alexa488 +Alexa546; -Alexa488 +Alexa546. All the sample preparations were analyzed before the active photobleaching FRET measurements. Under our experimental conditions, no significant background of Alexa 546 excitation in the absence of Alexa488 was observed. Furthermore, the test presented in Figure 2B (acceptor levels vs. E%) was weighted against the presence of crosstalk artifacts.

## Conclusions

Using confocal microscopy FRET [8] we have been able to detect the transfer of specific peptides into MHC II complexes at the membrane surface of mDC. Moreover, the profile of the appearance of MHC II - tumor lysate antigen complexes, as a function of time after the maturation stimulus, is in good agreement with the results from previous biological studies on mouse [9] and human DC [10]. In conclusion, our findings suggest that, in cancer vaccination immunotherapy procedures: i) autologous tumor lysates are correctly processed by DC *in vitro *and ii) the resulting antigenic peptides are properly loaded on mDCs' MHC II complexes. This study reinforces the rationale behind the immunogenic efficacy of cancer vaccination treatments.

## Abbreviations

ATL: Autologous Tumor Lysate; DC: Dendritic Cell; E%: Energy Transfer Efficiency; FRET: Förster Resonance Energy Transfer; HLA-DR: human leukocyte antigen DR; MHCII: major histocompatibility complex class II; ROI: Region of interest. SDS-PAGE: sodium dodecyl sulphate polyacrylamide gel electrophoresis.

## Competing interests

The authors declare that they have no competing interests.

## Authors' contributions

LF and VA carried out the ATL conjugation and cell sample preparations, participated in the study design and drafted the manuscript; TR participated in FRET measurements; MP, AMG, AR and EP performed *in vitro *culturing of dendritic cell vaccines; RR and LR performed the therapeutic treatments; JRK participated in analysis and interpretation of data and in manuscript revision; PN conceived the study, coordinated the groups, performed FRET measurements, and edited the manuscript. All authors read and approved the final manuscript.

## References

[B1] Hart DN Dendritic cells: unique leukocyte populations which control the primary immune response Blood 1997 90 3245 3287 9345009

[B2] Banchereau J Palucka AK Dhodapkar M Burkeholder S Taquet N Rolland A Taquet S Coquery S Wittkowski KM Bhardwaj N Pineiro L Steinman R Fay J Immune and clinical responses in patients with metastatic melanoma to CD34(+) progenitor-derived dendritic cell vaccine Cancer Res 2001 61 6451 6458 11522640

[B3] Ichim CV Revisiting immunosurveillance and immunostimulation: Implications for cancer immunotherapy J Transl Med 2005 3 8 10.1186/1479-5876-3-8 15698481 PMC549049

[B4] O'Neill DW Adams S Bhardwaj N Manipulating dendritic cell biology for the active immunotherapy of cancer Blood 2004 104 2235 2246 10.1182/blood-2003-12-4392 15231572

[B5] Banchereau J Palucka AK Dendritic cells as therapeutic vaccines against cancer Nat Rev Immunol 2005 5 296 306 10.1038/nri1592 15803149

[B6] Ridolfi R Petrini M Fiammenghi L Stefanelli M Ridolfi L Ballardini M Migliori G Riccobon A Improved overall survival in dendritic cell vaccination-induced immunoreactive subgroup of advanced melanoma patients J Transl Med 2006 4 36 10.1186/1479-5876-4-36 16914047 PMC1562447

[B7] Ridolfi R Riccobon A Galassi R Giorgetti G Petrini M Fiammenghi L Stefanelli M Ridolfi L Moretti A Migliori G Fiorentini G Evaluation of in vivo labelled dendritic cell migration in cancer patients J Transl Med 2004 2 27 10.1186/1479-5876-2-27 15285807 PMC509425

[B8] Bastiaens PIH Jovin TM Celis JE Fluorescence resonance energy transfer (FRET) microscopy Cell biology: a laboratory handbook 1998 3 New York, Academic Press 136 146

[B9] Turley SJ Inaba K Garrett WS Ebersold M Unternaehrer J Steinman RM Mellman I Transport of peptide-MHC class II complexes in developing dendritic cells Science 2000 288 522 527 10.1126/science.288.5465.522 10775112

[B10] Cella M Engering A Pinet V Pieters J Lanzavecchia A Inflammatory stimuli induce accumulation of MHC class II complexes on dendritic cells Nature 1997 388 782 787 10.1038/42030 9285591

[B11] Inaba K Turley S Iyoda T Yamaide F Shimoyama S Reis e Sousa C Germain RN Mellman I Steinman RM The formation of immunogenic major histocompatibility complex class II-peptide ligands in lysosomal compartments of dendritic cells is regulated by inflammatory stimuli J Exp Med 2000 191 927 936 10.1084/jem.191.6.927 10727455 PMC2193115

[B12] Vogel SS Thaler C Koushik SV Fanciful FRET Sci STKE 2006 2006 re2 10.1126/stke.3312006re2 16622184

[B13] Wallrabe H Elangovan M Burchard A Periasamy A Barroso M Confocal FRET microscopy to measure clustering of ligand-receptor complexes in endocytic membranes Biophys J 2003 85 559 571 10.1016/S0006-3495(03)74500-7 12829510 PMC1303111

[B14] Kenworthy AK Edidin M Distribution of a glycosylphosphatidylinositol-anchored protein at the apical surface of MDCK cells examined at a resolution of <100 A using imaging fluorescence resonance energy transfer J Cell Biol 1998 142 69 84 10.1083/jcb.142.1.69 9660864 PMC2133040

[B15] Ancarani V Fiammenghi L Petrini M Pancisi E Ridolfi L Ridolfi R Riccobon A Neyroz P Fluorescence microscopy imaging to monitor dendritic cell's tumor lysate capturing and processing: preliminary data [abstract] Italian J Biochem, Special Issue 2007 129

[B16] Unternaehrer JJ Chow A Pypaert M Inaba K Mellman I The tetraspanin CD9 mediates lateral association of MHC class II molecules on the dendritic cell surface Proc Natl Acad Sci USA 2007 104 234 239 10.1073/pnas.0609665104 17190803 PMC1765441

[B17] Gilboa E DC-based cancer vaccines J Clin Invest 2007 117 1195 1203 10.1172/JCI31205 17476349 PMC1857263

[B18] Lim TC Bailey VJ Ho Y-P Wang T-H Intercalating dye as an acceptor in quantum-dot-mediated FRET Nanotechnology 2008 19 075701 (7pp) doi: 10.1088/0957-4484/19/7/075701 10.1088/0957-4484/19/7/075701 21817649

